# Single‐Crystal‐to‐Single‐Crystal Transformation in a Copper(II) Complex: Structural and Spectroscopic Insights into Methanol Elimination and Hydrogen Bond Network Formation

**DOI:** 10.1002/open.202500126

**Published:** 2025-08-13

**Authors:** Babak Mirtamizdoust, Amirhossein Karamad, Faeze Mojtabazade, Hassan Hosseini‐Monfared, Rahman Bikas

**Affiliations:** ^1^ Inorganic Materials and Coordination Compounds Research Lab. Department of Chemistry Faculty of Science University of Qom Alghadir Street Qom 37161‐46611 Iran; ^2^ Department of Chemistry Faculty of Science University of Zanjan Daneshgah Boulevard Zanjan 38791‐45371 Iran; ^3^ Department of Chemistry Amirkabir University of Technology (Tehran Polytechnic) Hafez Street Tehran 34311‐15916 Iran; ^4^ Department of Chemistry Faculty of Science Imam Khomeini International University Qazvin‐Zanjan Fwy Qazvin 34148‐96818 Iran

**Keywords:** copper complexes, Hirshfeld surfaces, infrared spectroscopy, single‐crystal‐to‐single‐crystal, UV–vis, X‐ray diffraction

## Abstract

This study investigates the single‐crystal‐to‐single‐crystal (SCSC) transformations of a copper(II) complex, [Cu(L1)_2_(acac)_2_]·2CH_3_OH (**1**), with an octahedral coordination geometry. The complex is synthesized using L1 (6‐phenyl‐1,3,5‐triazine‐4,2‐diamine; C_9_H_18_N_5_) and copper acetylacetonate. In this structure, copper is coordinated to four oxygen atoms from two monoanionic acetylacetonate (acac) ligands and two nitrogen atoms from two neutral L1 ligands. The transformation of complex **1** into complex **2** is achieved by heating at 80 °C for 48 h, leading to the removal of methanol. This elimination facilitates the formation of direct hydrogen bonds between the NH_2_ groups and nitrogen atoms of adjacent triazine rings, establishing a network of intermolecular interactions. Structural analysis revealed a 0.2 Å elongation of the copper–nitrogen bond in the L1 ligand as a result of methanol removal. Complementary characterization techniques, including FTIR, UV–vis spectroscopy, and Hirshfeld surface analysis, are employed to further elucidate the transformations. The impact of methanol elimination on the crystal structure is assessed, highlighting the changes in intermolecular interactions.

## Graphical abstract

A methanol‐ligated copper(II) complex undergoes a single‐crystal‐to‐single‐crystal transformation upon solvent loss, retaining crystallinity while reorganizing its coordination geometry and hydrogen‐bonding network.

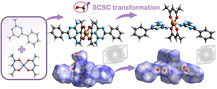

## 1 Introduction

Copper complexes are widely utilized across a range of applications, spanning from biological systems to industrial processes, due to their favorable redox properties, kinetic stability, and ability to undergo electron transfer reactions.^[^ ^1^, ^2^, ^3^, ^4^, ^5^, ^6^, ^7^, ^8^, ^9^, ^10^, ^11^, ^12^, ^13^, ^14^, ^15^, ^16^, ^17^ ^–^ ^18^ ^]^ Wang and colleagues explored the role of copper complexes in biological systems, such as DNA, where these complexes can cleave DNA in the presence of activators like H_2_O_2_, ascorbate, 3‐mercaptopropionic acid, and glutathione.^[^ ^19^, ^20^ ^–^ ^21^ ^]^ Additionally, copper complexes have been proposed as potential anticancer agents and have been employed in the synthesis of N‐heterocycles.^[^ ^22^ ^,^ ^23^ ^]^ Boni and coworkers utilized copper carbene and nitrene chemistry in organic synthesis, specifically focusing on enantioselective C–H functionalization reactions.^[^ ^24^ ^]^

Single‐crystal‐to‐single‐crystal (SCSC) transformations represent a class of crystal‐to‐crystal transitions in which the integrity and long‐range structural order of the crystal are maintained throughout the transformation process. These transformations are particularly intriguing because they can lead to materials with distinct properties, such as advanced adsorbents, magnetic switches, and responsive actuators.^[^ ^25^ ^,^ ^26^ ^]^ Crystal‐to‐crystal transformations can be triggered by various stimuli, including temperature, pressure, light, or chemical reactions. These transformations may occur through physical processes, where the crystal undergoes a temperature or pressure change, resulting in atomic rearrangements within the lattice. Alternatively, chemical transformations involve the rystal reacting with other compounds, leading to the formation of a new crystal structure with a distinct lattice arrangement.^[^ ^27^ ^,^ ^28^ ^]^ This study focuses on thermally triggered SCSC transformations.

## 2 Experimental Section

### 2.1 Materials and Instruments

The following chemicals were used in this study: ethanol (96%, Bidestan, Iran), methanol (99%, Merck), acetone (99%, Merck), tetrahydrofuran (Merck), 2‐hydroxy‐1,3‐diaminopropane (Alpha Acer), copper acetylacetonate (Merck), cyanuric chloride (Merck), phenyllithium (Sigma–Aldrich), and acetonitrile (Merck).

Infrared (IR) spectra of ligands and complexes were recorded after purification and preparation of KBr pellets using a Nicolet IS 10 and Bruker spectrometer, covering the 400–4000 cm^−1^ range. UV–vis spectra were obtained with a Helios Alpha Thermo Spectronic spectrophotometer. Melting points of ligands and complexes were determined using a Barnstead Electrothermal 9100 apparatus. For UV–vis analysis, solutions of the target compounds were prepared at a concentration of 1 × 10^−3^ M, and appropriate dilutions were made to obtain concentrations suitable for spectrometric measurements.

### 2.2 Preparation of the Complex [Cu(L1)_2_(acac)_2_]·2CH_3_OH (**1**)

Details regarding the synthesis of ligand L1 (C_9_H_18_N_5_) can be found in the Supporting Information. To prepare complex **1**, 0.06 g (0.32 mmol) of L1 and 0.12 g (0.48 mmol) of copper acetylacetonate were weighed and carefully placed at the bottom of a branched tube. Methanol (10 mL) was then added to the tube. The tube was sealed with a paraffin strip and immersed in a paraffin bath at 60 °C. After three days, light blue cubic crystals suitable for crystallization were isolated. The crystals were washed with acetone and ether and subsequently dried. The elemental analysis for complex **1** gave the following results: calculated for C_30_H_40_CuN_10_O_6_: C 51.46, H 5.76, N 22.00, and Cu 9.07%; found: C 51.r, H 5.71, N 21.98, and Cu 9.08%.

### 2.3 Preparation of the Complex [Cu(L1)_2_(acac)_2_] (**2**)

Complex **1** underwent transformation when placed in an oven at 80 °C for 48 h, during which it changed color from light blue to a deeper shade. The transformation was confirmed by various analytical techniques, including IR spectroscopy and X‐ray diffraction (XRD) crystallography. The elemental analysis for complex **2** was consistent with that of complex **1**, yielding the following results: calculated for C_28_H_32_CuN_10_O_4_: C 52.86, H 5.07, N 22.02, and Cu 9.99%; found: C 53.00, H 5.01, N 21.98, and Cu 10.08%.

### 2.4 IR and UV–vis Spectroscopy

**Figure 1:**
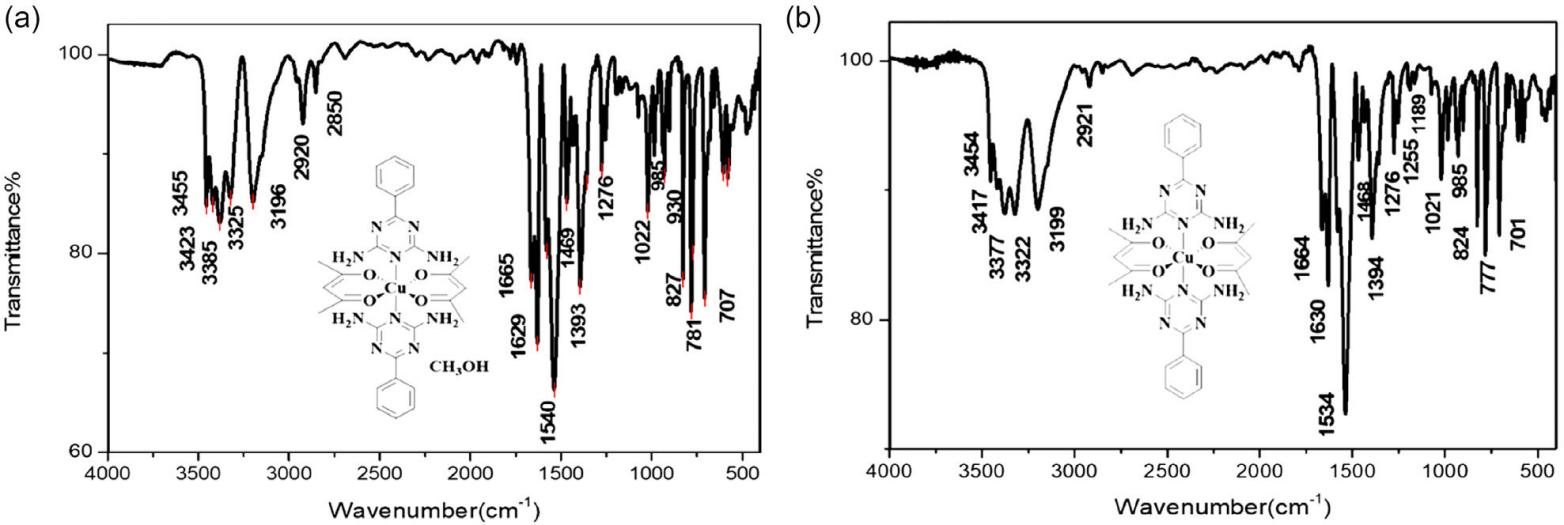
IR spectra of a) Complex **1** and b) Complex **2**.

The IR spectrum of the complex is presented in **Figure** **1**. The IR data further support the successful synthesis of the complex. The key absorption bands and their assignments are summarized in **Table** **1**, providing insights into the functional groups present in the complex structure.

**Table 1:** Key IR absorption bands and assignments for Complexes **1** and **2**.

	Complex **1**	Complex **2**
NH_2_ (stretching)	3385, 3325	3377, 3322
C=N	1629	1630
C—H (aliphatic)	2920	2921
C=C (aromatic)	1540	1534
C—H (aromatic, stretching)	3196	3199
NH_2_ (bending scissors)	1469	1468
OH (methanol)	3455	–
C=O	1665	1664
C—H	2850	–
C—N	1393	1394
C—H (aromatic, bending)	781	777
NH_2_ (off‐plate bending)	827	824

**Figure 2:**
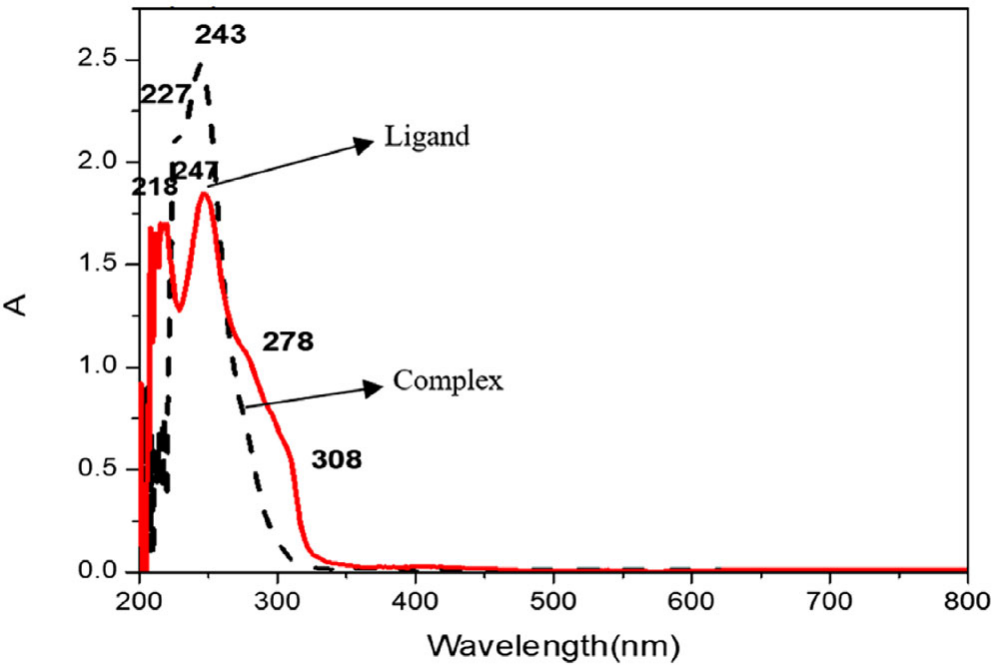
UV–vis absorption spectra.

Solution UV–vis spectra of complexes **1** and **2** were recorded in methanol (2 × 10^−5^ M, 200–800 nm) to investigate their electronic properties. However, these measurements primarily capture intraligand charge transfer (ILCT) and metal‐to‐ligand charge transfer (MLCT) transitions and are limited in detecting d–d transitions, which are highly sensitive to the coordination environment of the metal center. In solution, the spectra of complexes **1** and **2** are nearly identical (**Figure** **2**), likely due to solvent interactions masking differences in their coordination environments. These solution‐based data are therefore less informative for characterizing the electronic structure changes relevant to this study and have been excluded from the main text.

Notably, a visible color change from light blue to a deeper shade was observed upon heating complex **1**, suggesting a modification in the coordination environment that likely affects the d–d transitions. Diffuse reflectance spectroscopy (DRS) would be ideal for probing these transitions in the solid state, as it preserves the coordination environment and is better suited to detect the weak d–d bands responsible for the observed color change. Due to current equipment limitations, DRS measurements could not be performed. However, the observed color change aligns with reported behavior in similar [metal/ligand] systems, where DRS studies have identified d–d transitions in the [X–Y nm] range corresponding to [geometry/ligand field changes].^[^ ^29^ ^]^ Complementary characterization data provide further evidence of the structural and electronic differences between complexes **1** and **2**. Future studies will prioritize DRS to directly characterize the d–d transitions associated with the observed color change.

### 2.5 Thermal Analysis

**Figure 3:**
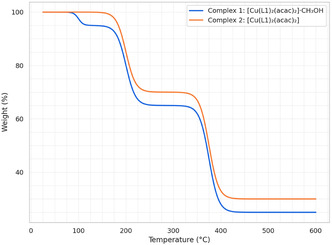
TGA of Complexes **1** and **2**.

To further support the SCSC transformation and investigate the thermal stability of the complexes, thermogravimetric analysis (TGA) was performed on both Complex **1** and Complex **2** (**Figure** **3**).

Complex **1** shows a distinct three‐step decomposition pattern. The first mass loss occurs in the range of 80–150 °C and corresponds to the release of lattice methanol. This thermal event confirms the initial step in the SCSC process, as methanol elimination triggers the structural rearrangement and hydrogen bond reorganization. The second and third decomposition steps, occurring between 150–500 °C, are attributed to the stepwise breakdown of the organic ligands (L1 and acac). After 500 °C, the residue stabilizes, which is consistent with the formation of copper(II) oxide (CuO).

In contrast, Complex **2** shows no weight loss below 150 °C, confirming the absence of methanol. Its decomposition follows a similar two‐step pattern corresponding to ligand degradation and ends with a comparable CuO residue.

The TGA results clearly support the occurrence of the SCSC transformation and provide evidence of structural changes initiated by methanol removal. The overall thermal behavior of both complexes is consistent with structurally related copper(II) systems reported in the literature.

### 2.6 X‐ray Diffraction (XRD) Analysis

Single crystals of the compounds were mounted on a glass fiber, lubricated, and subsequently cooled to −93 °C using a nitrogen gas stream controlled by the Cryostream Controller 700. XRD data were collected using a Bruker SMART APEX II X‐ray diffractometer with graphite‐monochromated Mo K*α* radiation (*λ *= 0.71073 Å). The instrument was operated at 50 kV and 30 mA. Data were collected within a 2*θ* range of 8.08° to 52.00°. No significant decay in intensity was observed during the data collection process.

Data processing was performed using the Bruker AXS Crystal Structure Analysis Package.^[^ ^30^, ^31^, ^32^ ^–^ ^33^ ^]^ The following steps were employed: Data collection using APEX2, unit cell refinement and data reduction with SAINT, absorption correction via SADABS, structure solution with XPREP and SHELXS‐97, and structure refinement using SHELXL‐97. Molecular graphics and publication material preparation were done with SHELXTL.^[^ ^34^ ^]^ The neutral atom scattering factors were derived from Cromer and Waber's tables.^[^ ^35^ ^]^ The crystal space group was determined based on systematic absences, E‐statistics, and successful structure refinement. The structure was solved using direct methods, and full‐matrix least‐squares refinements were performed on the compounds, minimizing the function ∑*w*(Fo^2^ − Fc^2^)^2^ (**Table** **2**).

**Table 2:** Crystallographic data and refinement parameters.

Compound	Complex **1**	Complex **2**
CCDC Number	2278384	2278386
Empirical formula	C_28_H_32_CuN_10_O_4_·2(CH_4_O)	C_28_H_32_CuN_10_O_4_
Formula weight	700	636.18
Temperature [K]	120	293(2)
Crystal system	Triclinic	Triclinic
Space group	Pī	Pī
*a* [Å]	8.1133 (3)	10.1175(5)
*b* [Å]	9.4465 (4)	10.8768(5)
*c* [Å]	12.1864 (4)	14.7096(8)
*α* [°]	67.226 (4)	76.989(4)
*β* [°]	83.390 (3)	71.661(5)
*γ* [°]	73.572 (4)	85.401(4)
Volume [Å^3^]	826.00 (6)	1497.00(13)
*Z*	1	2
*ρ*_calc_mg [mm^3^]	1.638	1.411
*m* [mm^−^^1^]	1.43	0.781
F (000)	367	662.0
Crystal size [mm^3^]	0.36 × 0.21 × 0.16	0.50 × 0.40 × 0.30
2*θ* range for data collection	0.705–0.843	5.76–55°
Index ranges	−8 ≤ *h* ≤ 9, −11 ≤ *k *≤ 11, −14 ≤ *l* ≤ 14	−13 ≤ *h *≤ 12, −13 ≤ *k* ≤ 14, −18 ≤ *l *≤ 19
Reflections collected	3355	21,429
Independent reflections	2929 [R(int) = 0.019]	6844[R(int) = 0.0200]
Data/restraints/parameters	2929/0/230	6844/0/389
Goodness‐of‐fit on F^2^	1.068	0.954
Final R indexes [I >= 2*σ* (I)]	R_1_ = 0.0295, wR_2_ = 0.0913	R_1_ = 0.0351, wR_2_ = 0.0909
Final R indexes [all data]	R_1_ = 0.0318, wR_2_ = 0.0940	R_1_ = 0.0593, wR_2_ = 0.0955
Largest diff. peak/hole [eÅ^−3^]	0.38, −0.32	0.26, −0.30

## 3 Results and Discussion

**Scheme 1:**
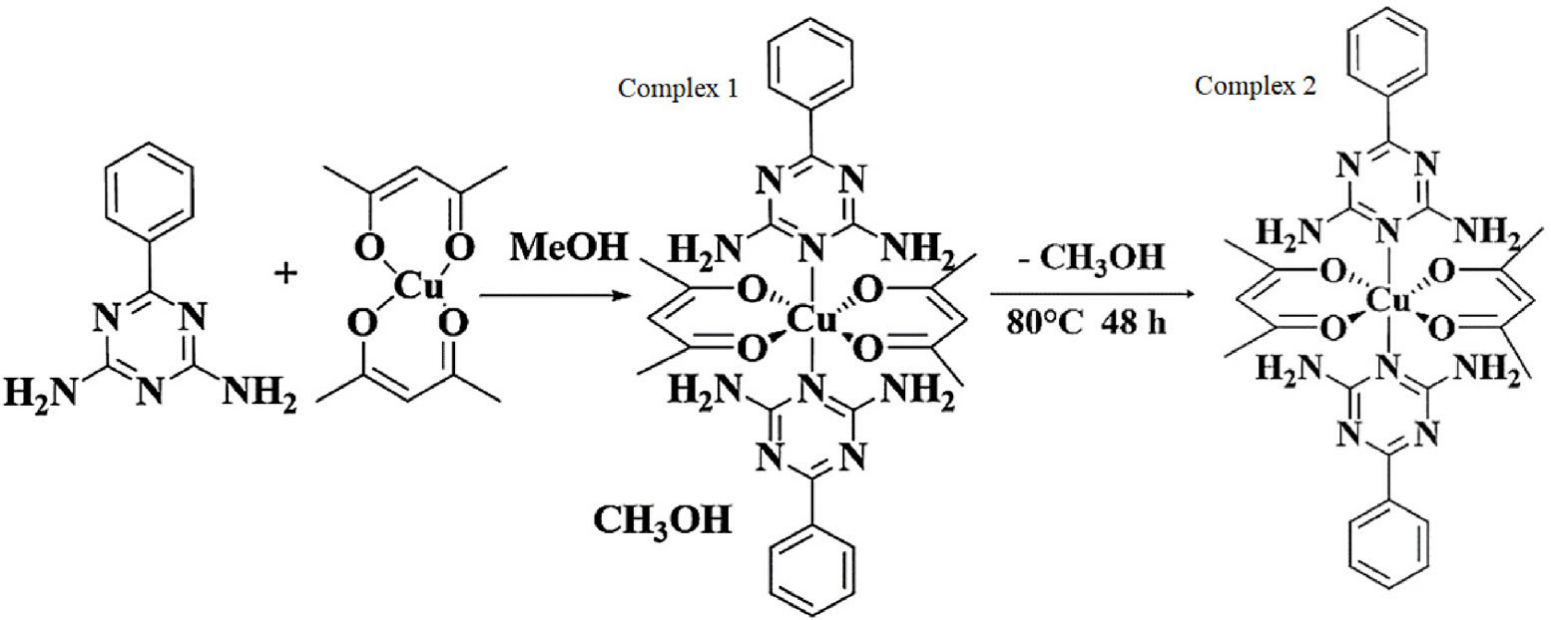
Synthesis pathway of the complexes.

The complex [Cu(L1)_2_(acac)_2_]·2CH_3_OH (**1**) was synthesized through the reaction of L1 and copper acetylacetonate in a 1:2 molar ratio in methanol solvent. The resulting complex exhibited a melting point of 235 °C. Upon removal of methanol, Complex **1** underwent a transformation to [Cu(L1)_2_(acac)_2_] (**2**), which no longer contained methanol. This process, along with the impact of methanol removal, will be discussed in detail. The transformation was characterized by a shift in the color of the crystals from light blue to navy blue, although the crystal structure remained unchanged. The newly obtained crystals demonstrated good quality, enabling their structure to be determined via XRD.^[^ ^36^ ^]^ The melting point of the anhydrous complex was found to be 240 °C (**Scheme** **1**).

**Figure 4:**
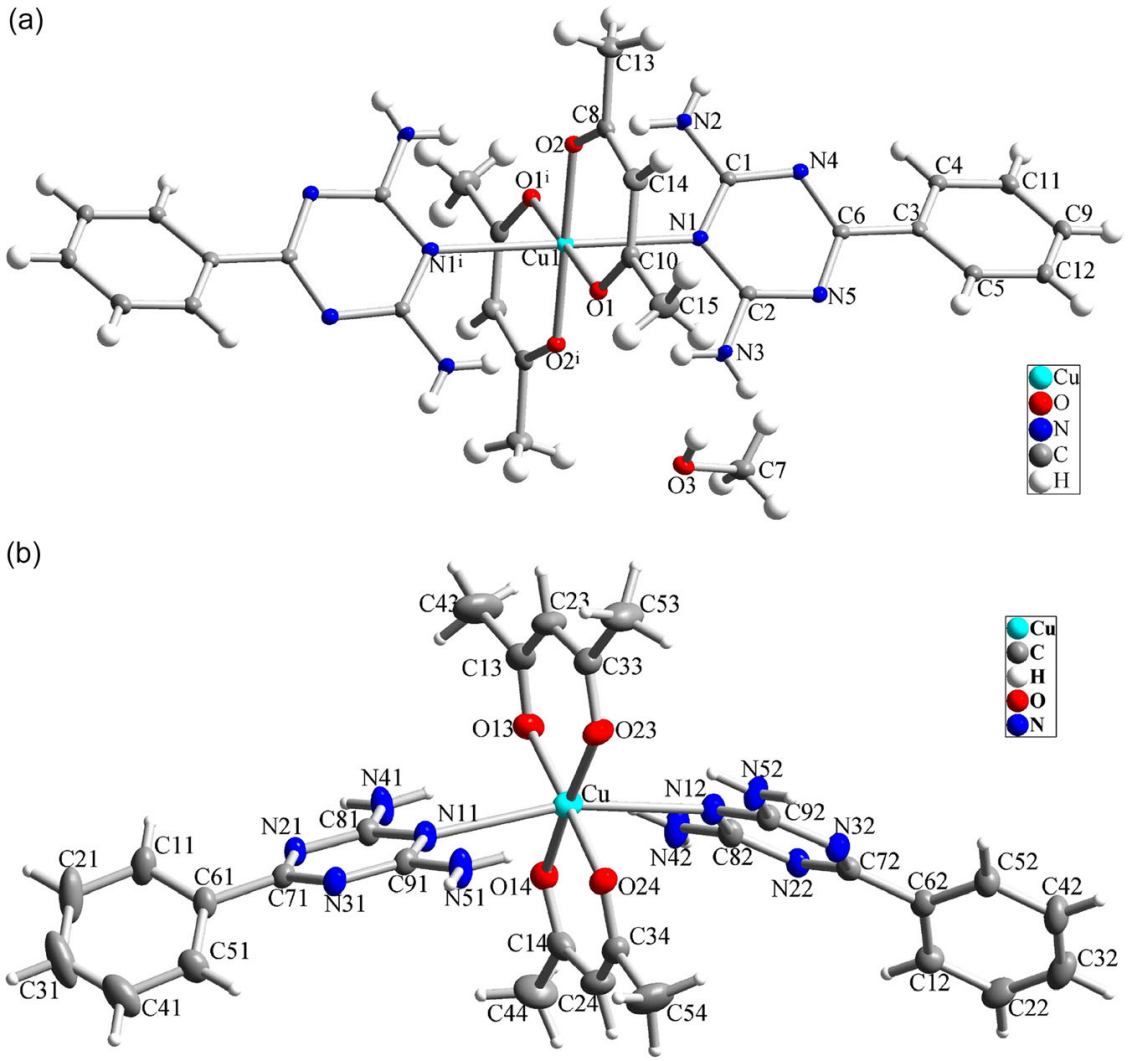
Molecular structure of a) Complex **1** and b) Complex **2**; ellipsoids are drawn at the 30% probability level.

**Figure 5:**
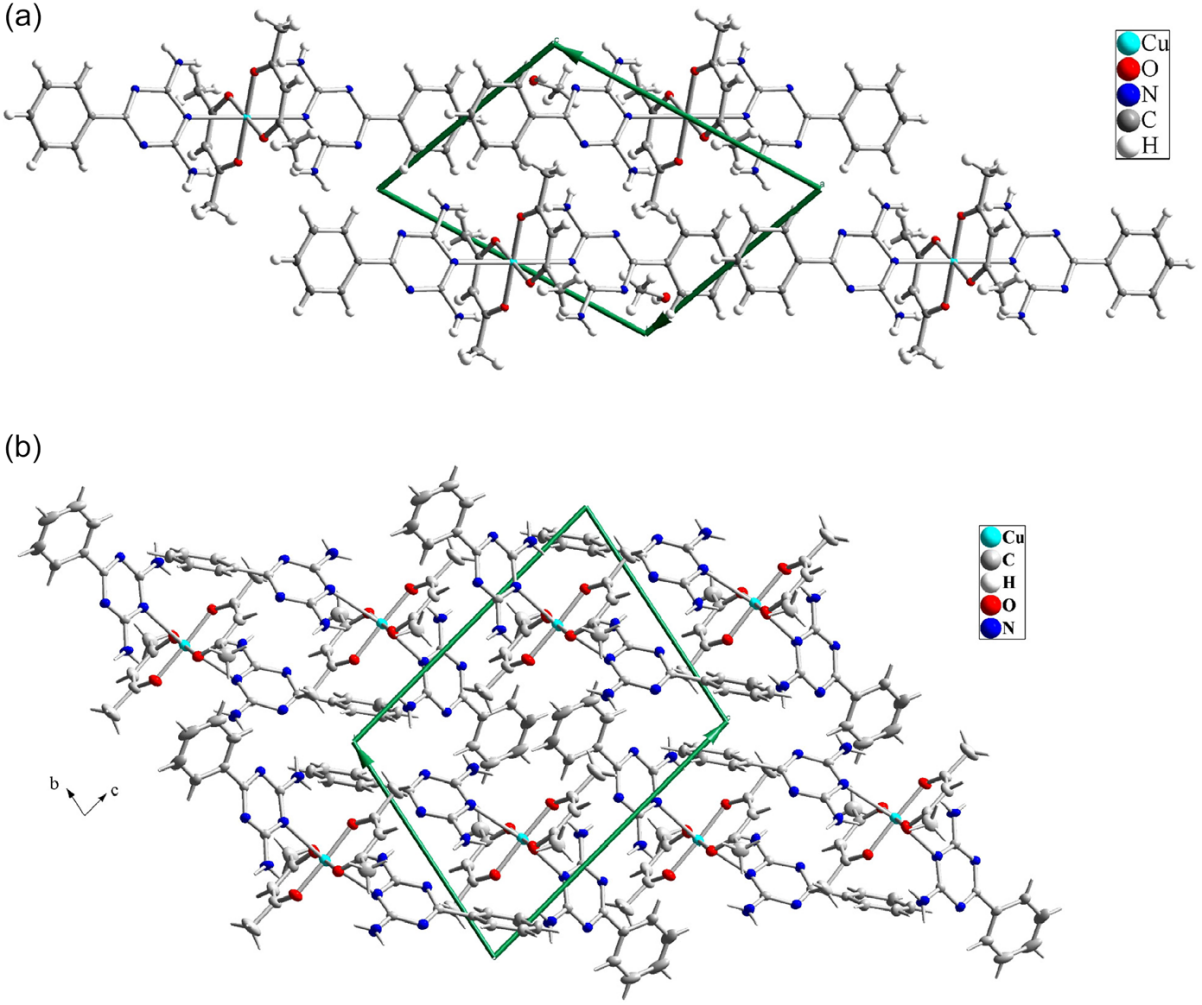
Crystal lattice of a) Complex **1** and b) Complex **2**.

The crystal structure of Complex **1** was determined by XRD, revealing that it crystallized in the triclinic crystal system with the space group Pī. These crystallographic parameters are also applicable to Complex **2**. The crystal structures of both complexes are depicted in **Figure** **4**, while the crystal lattice along the a‐axis is shown in **Figure** **5**. Selected bond lengths and angles are provided in **Table** **3**.

**Table 3:** Selected bond lengths and bond angles.

Bond	Length [Ǻ]	Bond	Angle [°]
Complex **1**			
Cu1—O1	1.9330 (10)	O1—Cu1—O1^i^	180.0 (5)
Cu1—O1^i^	1.9330 (10)	N1—Cu1—N1^i^	180.0 (5)
Cu1—N1^i^	2.550 (4)	O1—Cu1—O2	93.64 (5)
Cu1—O2	1.9498 (12)	O1—Cu1—O2^i^	86.36 (5)
O1—C10	1.272 (2)	O1^i^—Cu1—O2	86.36 (5)
O2—C8	1.266 (2)	O1^i^—Cu1—O2^i^	93.64 (5)
O3—C7	1.423 (2)	O2—Cu1—O2^i^	180.0 (5)
N1—C1	1.3445 (19)	Cu1—O1—C10	124.37 (9)
N1—C2	1.3454 (16)	Cu1—O2—C8	124.43 (8)
N2—C1	1.3328 (17)		
N3—C2	1.336 (2)		
Complex **2**			
Cu—O14	1.9227 (14)	C71—N31	1.334 (2)
Cu—N11	2.7062 (14)	Cu—N12	2.7512 (2)
Cu—O24	1.9235 (13)	C71—N21	1.334 (2)
Cu—O23	1.9242 (14)	C81—N41	1.329 (2)
Cu—O13	1.9277 (14)	C81—N11	1.338 (2)
C13—O13	1.268 (2)	C81—N21	1.356 (2)
C13—C23	1.394 (3)	C91—N51	1.333 (2)
C13—C43	1.505 (3)	C91—N11	1.340 (2)
C23—C33	1.380 (3)	C91—N31	1.359 (2)
C33—O23	1.263 (2)	C12—C62	1.382 (3)
C33—C53	1.517 (3)	C12—C22	1.384 (3)
C14—O14	1.265 (2)	C22—C32	1.357 (3)
C14—C24	1.378 (3)	C32—C42	1.371 (3)
C14—C44	1.513 (3)	C42—C52	1.385 (3)
C24—C34	1.386 (3)	C52—C62	1.381 (2)
C34—O24	1.274 (2)	C62—C72	1.488 (3)
C34—C54	1.508 (3)	C72—N22	1.331 (2)
C11—C61	1.385 (3)	C72—N32	1.333 (2)
C11—C21	1.388 (3)	C82—N42	1.335 (2)
C21—C31	1.377 (4)	C82—N12	1.346 (2)
C31—C41	1.363 (4)	C82—N22	1.353 (2)

Symmetry code: (i) −*x* + 1, −*y*, −*z* + 1.

In both complexes, each copper atom is coordinated by two nitrogen atoms from two neutral 6‐phenyl‐1,3,5‐triazine‐2,4‐diamine (L1) ligands and four oxygen atoms from two anionic acac ligands. However, due to the removal of methanol and subsequent transformation, the Cu—N bond lengths increase from 2.550(4) Å in Complex **1** to 2.7512(2) Å and 2.7062(14) Å in Complex **2**. Conversely, the Cu—O bond lengths decrease from 1.9330(10) and 1.9498(12) Å in Complex **1** to 1.9227(14), 1.9235(13), 1.9242(14), and 1.9277(14) Å in Complex **2**. Consequently, in both complexes, the copper cation maintains an oxidation state of +2, and the coordination environment is described as *trans*‐CuN_2_O_4_.

In the crystal structures of both complexes, the coordination sphere around each copper atom is distorted octahedral. Two L1 ligands coordinate through the nitrogen atoms of the triazine ring in the axial positions, while two acac ligands coordinate through their oxygen atoms in the equatorial positions. In both complexes, the bond lengths involving the L1 ligands are longer than those of the other ligands, indicating a significant Jahn–Teller distortion along these ligands. Notably, in Complex **2**, there is a substantial increase in the Cu—N bond length of the L1 ligand, with a 0.2 Å elongation compared to Complex **1**. Furthermore, the removal of methanol from the crystalline lattice causes the L1 ligand to bend, resulting in an arc‐like shape for the complex.

**Figure 6:**
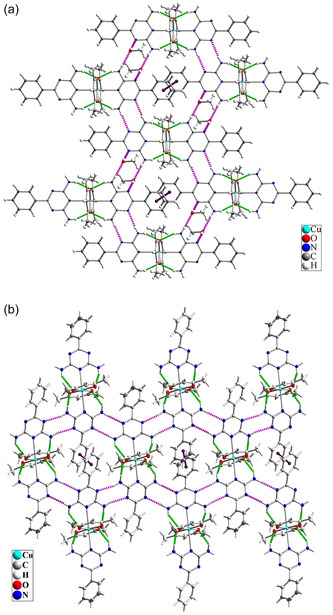
Intermolecular hydrogen bonding and π–π stacking interactions in the crystal structure of a) Complex **1** and b) Complex **2**.

In the crystal lattice of Complex **1**, various noncovalent interactions are present, including intramolecular hydrogen bonding, intermolecular hydrogen bonding, and π–π stacking interactions. Intermolecular hydrogen bonding occurs between the N–H groups of L1 ligands and the nitrogen atoms of the triazine rings of adjacent molecules within the crystalline lattice. Two methanol molecules are located adjacent to the complex, where they are connected to the complex via intermolecular hydrogen bonding. The presence of methanol plays a crucial role in the crystal lattice and the nature of the intermolecular hydrogen bonding interactions in Complex **1**. Methanol molecules interact with the nitrogen atoms of the L1 ligands on one side and with the NH_2_ groups of adjacent L1 ligands via hydrogen bonding on the other side, effectively linking two adjacent molecules. These interactions result in the formation of a polymeric lattice structure, creating a 2D grid with repeating hexagonal units. In both Complex **1** and Complex **2**, intramolecular hydrogen bonding is formed between the N–H groups of L1 ligands and the coordinated oxygen atoms of the acac ligands. However, upon the removal of methanol molecules in Complex **2**, the arrangement of hydrogen bonds is altered. Specifically, intermolecular hydrogen bonding is reorganized, with direct hydrogen bonding forming between the NH_2_ groups of adjacent molecules and the nitrogen atoms of the triazine rings, eliminating the methanol intermediate (**Figure** **6**). **Table** **4** summarizes the hydrogen bonding parameters.

**Table 4:** Hydrogen bonding interactions.

D–H···A	D–H [Å]	H···A [Å]	D···A [Å]	D–H···A [°]
Complex **1**				
O3–H1o3···N4^i^	0.81 (3)	2.13 (3)	2.904 (2)	161 (2)
N3–H1n3···N5^ii^	0.757 (17)	2.298 (17)	3.0537 (16)	176 (3)
N2–H1n2···O1^iii^	0.83 (2)	2.37 (3)	3.072 (2)	143 (2)
N2–H1n2···O2	0.83 (2)	2.46 (2)	3.1076 (18)	135.5 (19)
N2–H2n2···O3^iv^	0.79 (2)	2.14 (2)	2.9237 (19)	170 (2)
N3–H2n3···O1	0.81 (2)	2.27 (2)	2.9574 (18)	143 (2)
Complex **2**				
N41–H41A···O13	0.86	2.15	2.938 (2)	151.9
N41–H41A···O14	0.86	2.53	3.111 (2)	126.1
N41–H41B···N21^i^	0.86	2.21	3.070 (2)	172.5
N51–H51A···O24	0.86	2.34	3.069 (2)	143.1
N51–H51B···N22^ii^	0.86	2.36	3.179 (2)	158.6
N42–H42A···O14	0.86	2.51	3.058 (2)	122.0
N42–H42A···O14	0.86	2.51	3.058 (2)	122.0
N42–H42B···N31^iii^	0.86	2.34	3.195 (2)	171.7
N52–H52A···O23	0.86	2.15	2.988 (2)	165.1
N52–H52B···N32^iv^	0.86	2.27	3.086 (2)	159.5

Symmetry code(s): (i) −*x* + 1, −*y* + 2, −*z* + 1; (ii) *x*, *y* + 1, *z*; (iii) *x*, *y−*1, *z*; (iv) −*x* + 2, −*y* + 1, −*z* + 2.

Complex **2** exhibited changes in its lattice parameters, with all bond lengths increasing after the removal of methanol. The most significant change was observed in the *c*‐axis, which increased from 12.1864(4) Å in Complex **1** to 14.7096(8) Å in Complex **2**. Additionally, notable changes occurred in the unit cell angles. The angle between the *b* and *c* axes (*β*) decreased significantly by 11.729°, while the other angles exhibited an increase.

**Figure 7:**
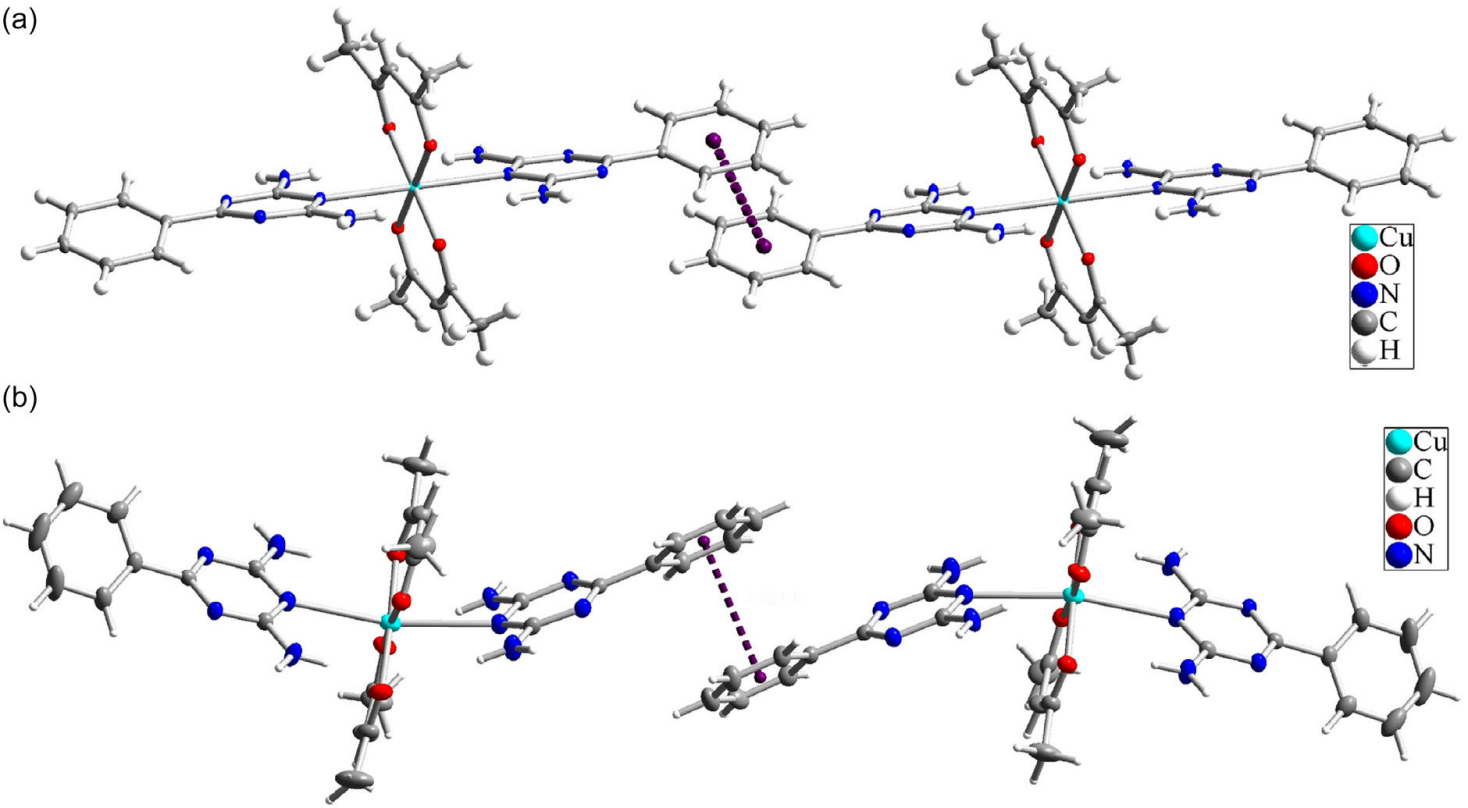
Comparison of π–π interactions and the arrangement of the L1 ligand in the crystals of a) Complex **1** and b) Complex **2**.

In the crystal lattice of Complex **1**, there is a strong π–π interaction between the phenyl rings attached to the triazine ring in the L1 ligand. However, in Complex **2**, this interaction weakens upon methanol removal and exhibits a more moderate intensity. As shown in **Figure** **7**, the distance between the centers of the phenyl rings in Complex **1** is 3.205 Å, which is considered a very strong π–π interaction according to published literature.^[^ ^37^ ^]^ In Complex **2**, this distance increases to 3.921 Å, reflecting a weaker interaction. Based on the literature, this interaction in Complex **2** is categorized as either moderately strong or weak.

The Hirshfeld surface (HS) concept was developed to define the volume occupied by a molecule within a crystal and to partition the electron density of the crystal into fragments corresponding to the molecular electron densities. HS analysis is a valuable tool for quantifying the nature and strength of intermolecular interactions. In this study, HS calculations were carried out using Crystal Explorer21.^[^ ^38^ ^]^

**Figure 8:**
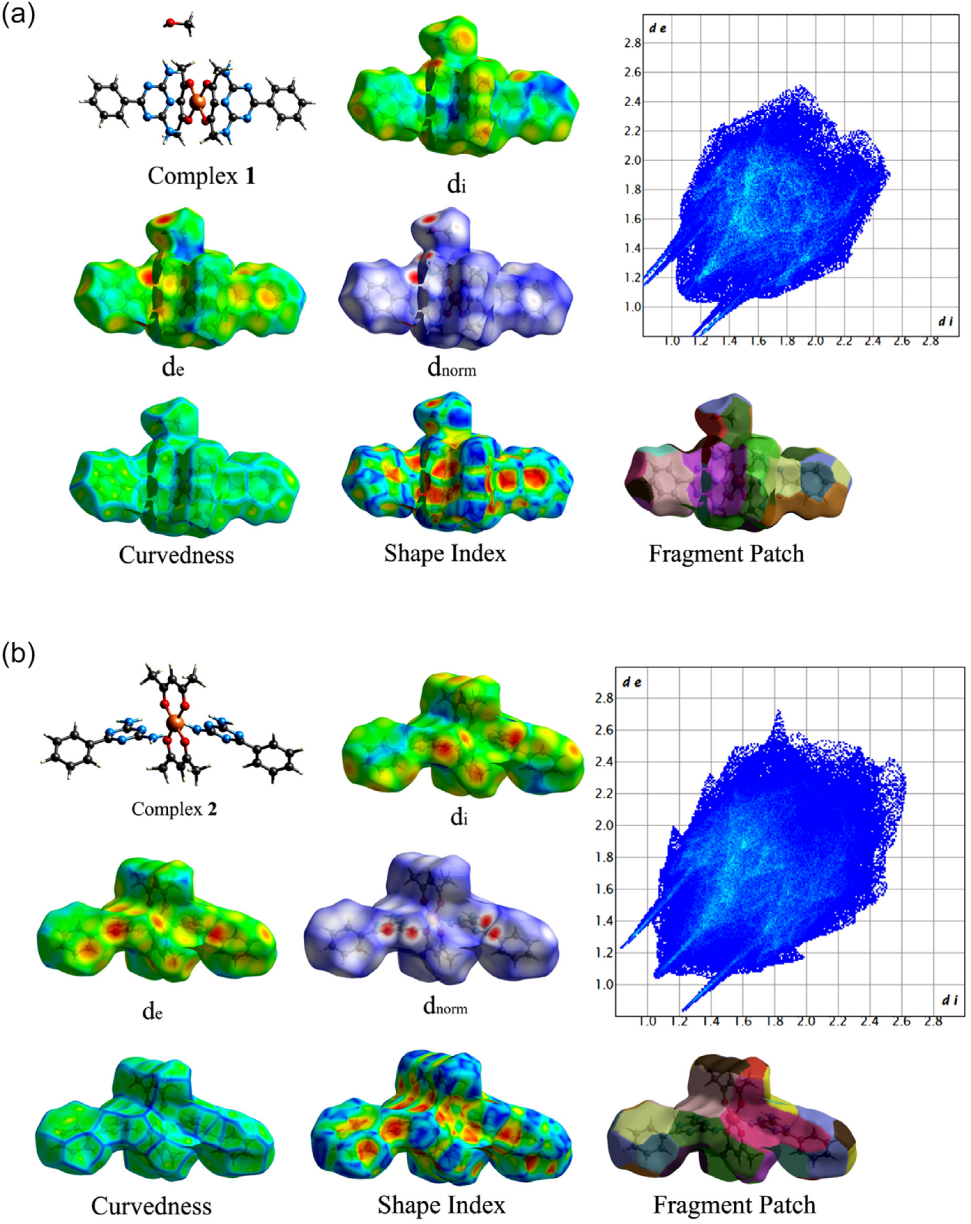
HS of a) Complex **1** and b) Complex **2**.

All Hirshfeld properties are shown in **Figure** **8**. The dnorm property is used to highlight blue areas, indicating that the contact distance between atoms inside and outside the surface is greater than the sum of their respective van der Waals radii. Conversely, small red regions indicate that the contact distance between atoms inside and outside the surface is less than the sum of their van der Waals radii. The white areas correspond to regions where the contact distance equals the sum of the van der Waals radii.^[^ ^39^ ^]^ In Figure 8, the dnorm property reveals that the oxygen of methanol in Complex **1** exhibits numerous red areas due to hydrogen bonding interactions. However, after the removal of methanol, these red areas are predominantly located around the copper center (Figure 6b).

## 4 Conclusion

This study focused on SCSC transformations, where Complex **1** was synthesized using L1 and copper acetylacetonate, resulting in an octahedral geometry. The complex was then heated at 80 °C for 48 h, resulting in the formation of Complex **2** and the removal of methanol. Both complexes feature two nitrogen atoms from the neutral 6‐phenyl‐1,3,5‐triazine‐2,4‐diamine (L1) ligands and four oxygen atoms from two monoanionic acac ligands. Both complexes crystallized in the space group Pī, with copper in the +2 oxidation state. The synthesis of the complexes was confirmed by IR spectroscopy. The removal of methanol led to structural changes, including a 0.2 Å increase in the bond length between the L1 nitrogen and the copper atom in Complex **2**, compared to Complex **1**. HS analysis revealed that, after methanol removal, the red regions were primarily located around the copper center.

## Conflict of Interest

The authors declare no conflict of interest.

## Data Availability

The data that support the findings of this study are available from the corresponding author upon reasonable request.
